# New insights into coupling and uncoupling of cerebral blood flow and metabolism in the brain

**DOI:** 10.3325/cmj.2016.57.223

**Published:** 2016-06

**Authors:** Poornima Venkat, Michael Chopp, Jieli Chen

**Affiliations:** 1 Department of Neurology, Henry Ford Hospital, Detroit, MI, USA; 2 Department of Physics, Oakland University, Rochester, MI, USA

## Abstract

The brain has high metabolic and energy needs and requires continuous cerebral blood flow (CBF), which is facilitated by a tight coupling between neuronal activity, CBF, and metabolism. Upon neuronal activation, there is an increase in energy demand, which is then met by a hemodynamic response that increases CBF. Such regional CBF increase in response to neuronal activation is observed using neuroimaging techniques such as functional magnetic resonance imaging and positron emission tomography. The mechanisms and mediators (eg, nitric oxide, astrocytes, and ion channels) that regulate CBF-metabolism coupling have been extensively studied. The neurovascular unit is a conceptual model encompassing the anatomical and metabolic interactions between the neurons, vascular components, and glial cells in the brain. It is compromised under disease states such as stroke, diabetes, hypertension, dementias, and with aging, all of which trigger a cascade of inflammatory responses that exacerbate brain damage. Hence, tight regulation and maintenance of neurovascular coupling is central for brain homeostasis. This review article also discusses the waste clearance pathways in the brain such as the glymphatic system. The glymphatic system is a functional waste clearance pathway that removes metabolic wastes and neurotoxins from the brain along paravascular channels. Disruption of the glymphatic system burdens the brain with accumulating waste and has been reported in aging as well as several neurological diseases.

The brain’s high energy requirements and limited storage capacity make persistent cerebral blood flow (CBF) critical for its proper functioning as well as for the prevention of damage and death. This explains why, in spite of constituting only about 2% of the total body weight, the brain easily commands about 20% of the total cardiac output as CBF (1).There are several protective and defense mechanisms in place to ensure adequate cerebral perfusion. One such mechanism is cerebral auto-regulation, which seeks to maintain constant CBF. Second, the arteries supplying the brain, namely the internal carotid arteries and vertebral arteries, which merge to form the basilar artery, are arranged into the “circle of Willis,” creating collaterals in the cerebral circulation. The circle of Willis enables shifting of blood flow from one brain hemisphere to the other, reversal of blood flow direction between carotid and basilar arterial systems, and diversion of circulation across extra cranial-intracranial boundaries (2). These defense mechanisms were believed to protect against a drop in CBF, ie, if an artery supplying the circle is blocked, blood flow from the other blood vessels is able to compensate and sustain sufficient CBF; and extend protection to the brain from hemodynamic stress and blood pressure fluctuations (3). However, the extent of protection provided by the circle of Willis against a decline in CBF is still questioned, as the communicating arteries are relatively small or hypoplastic in majority of the population, making them ineffective for blood transfer (3).

CBF-metabolism coupling is typically studied at two levels: in terms of total cerebral perfusion at the whole brain level and local and regional CBF changes in response to neuronal activation/stimulation. Depending on neuronal activation and metabolic demands, regions of the brain may be either hypo-perfused or hyper-perfused. A sudden decrease in CBF (such as during the occlusion of a cerebral artery) results in an ischemic stroke with neurological deficits, tissue damage, and even death. An excess of blood flow leads to hyperemia and, possibly, intracranial pressure increases leading to tissue compression and damage. Therefore, it is essential to maintain brain homeostasis, and neurovascular coupling maintains a balance between neuronal activity and subsequent CBF changes. The neurovascular unit is a conceptual model encompassing the anatomical and metabolic interactions between the neurons, vascular components (endothelial cells, pericytes, vascular smooth muscle cells) and glial cells (astrocytes and microglia) in the brain (4,5).While the neurovascular unit strives to ensure continuous supply of oxygen, glucose, and other nutrients to the brain, the glymphatic system targets waste removal such as metabolic byproducts and neurotoxins along paravascular channels (6). Water homeostasis is mediated by integral membrane pore proteins called aquaporins, which transport and regulate water movement in the brain. The roles and importance of the glymphatic system and water channels are also discussed in this review article.

## Neuronal coupling to CBF and metabolism

Studying chemical mediators of neurovascular coupling has been of immense research interest over the past several decades and continues to be investigated as no clear understanding or conclusions have been reached. Neural activation triggers hemodynamic responses resulting in vasodilation and increased CBF. The chemical mediators of neurovascular coupling are thought to function as *post hoc* mechanisms or occur in anticipation or in parallel with neural activity. These mediators maybe metabolic byproducts of neural and glial metabolism with vasodilator properties such as adenosine, nitric oxide (NO), ions like hydrogen (H^+^), potassium (K+), calcium (Ca^2+^), and lactate. NO is a powerful vasodilator and can be produced by neurons, glial cells, vascular cells, and endothelial cells lining the cerebral vessels (7). In the hippocampus, direct and simultaneous *in vivo* measurements of NO and CBF changes revealed that neurovascular coupling is mediated by diffusion of NO between active glutamatergic neurons and blood vessels (8).During brain activation and metabolism, lactate produced may mediate functional hyperemia via increasing H^+^ concentration and producing vasodilation (9,10). It still remains to be answered what these mediators are and whether they are *post hoc* mechanisms or occur in anticipation or in parallel with neural activity. Since most of these mediators may not have sufficiently high spatial and temporal resolution to function independently, it is likely that there is an interrelationship between several mediators and their pathways to effectively maintain neurovascular coupling.

## Role of astrocytes in CBF metabolism coupling

Astrocytes are a type of glial cell that are about five times as abundant as neurons in vertebrates (11).Their anatomical position places them at a strategic advantage to detect synaptic activation and couple it with glucose uptake, ie,1) astrocytic endfeet form a continuous layer surrounding cerebral blood vessels, 2) the processes of many vasoactive interneuron’s synapse onto astrocytes rather than directly onto blood vessels, 3) astrocytic processes ensheath synaptic contacts and neurotransmitter receptors, and 4) neurotransmitter receptor uptake sites are expressed on astrocytes (11-13).While the exact molecular mechanisms and messengers that relay neural activity to blood vessels are not understood, recent studies suggest a novel role for astrocytes in communicating neuronal activity levels to blood vessels and coordinating energy demand with oxygen and glucose supply (14). Astrocytic mediation of CBF-metabolism coupling may follow two possible scenarios: 1) changes in neuronal activity drive changes in energy metabolism, and astrocytes induce vasodilation or vasoconstriction by calcium-dependent release of vasoactive substances to alter CBF (15) and 2) changes in neuronal activity drive CBF and energy metabolism by inducing vasodilation or vasoconstriction by feed-forward mechanisms releasing neurotransmitter and neuropeptide molecules related to neuronal signaling (15). Astrocytes can mediate the synthesis and release of vasoactive gliotransmitters in response to neurotransmitters and neuropeptides (16,17). Neuroglial metabolic coupling is thought to regulate brain metabolism (16,17). Glial cells absorb glutamate from the synaptic cleft, and the byproduct of glial glycolysis, which is lactate, is consumed by neurons and metabolized. However, since the brain during rest and activation is usually supplied with much more glucose than is required, lactate as a major secondary fuel is required in critical circumstances such as hypoglycemia or strenuous exercise (18). Lactate produced by astrocytes is either locally oxidized, or selectively diffused to astrocytic endfeet to increase CBF (18). *In vivo* and *in vitro* data suggest that cortical astrocytes maintain a steady state reservoir of lactate that is immediately mobilized via a small rise in potassium channel during neuronal activation (19).

## Neurovascular uncoupling in neurological disease state

Neurovascular coupling describes the relationship between local neural activation and resultant CBF changes. The CBF changes are governed by changes in neural activity through a complex network of coordinated mechanisms involving neurons, glial cells, and vascular components. In the aging brain, as well as under neurological disease states such as ischemic stroke, traumatic brain injury, epilepsy, dementia, hypertension, diabetes mellitus, and glioma, neurovascular uncoupling may ensue (4,20,21). Neurovascular uncoupling can result from abnormalities in its chemical mediators as well as alterations in the vascular dynamics. After stroke, inflammatory cytokines such as tumor necrosis factor (TNF-alpha), interleukin (IL)-1, IL-6 and IL-12 have been implicated in the cascade leading up to BBB disruption and neurovascular uncoupling (22-24). As a result of neurovascular uncoupling, the blood brain barrier (BBB) integrity is compromised. The BBB is composed of microvascular endothelial cells connected by tight junctions, a thick basement membrane, and astrocytic endfeet. The BBB functions as a highly selective permeability membrane that provides a biochemical shield protecting the brain from the invasion of neurotoxins. When the BBB is permeable, it also allows infiltration of inflammatory cells and pro-inflammatory factors. Neurovascular uncoupling can ultimately lead to mitochondrial dysfunction and oxidative stress, neuronal death, and brain tissue atrophy (25,26). The vascular components play an important role in neurovascular coupling. For instance, endothelial cells can regulate vascular tone by releasing potent vasoactive factors (7). Vascular tone is determined by the contractile activity of smooth muscle cells that line the vessel walls, and largely affects the resistance to blood flow through the circulation (27). Under neurological disease states, BBB dysfunction and endothelial damage decrease vasodilation induced by the endothelium, and via the release of endothelin, which can induce vascular contraction (7). The ionic channels on vascular smooth muscle cells can also be altered in hypertension, diabetes, and dementia, leading to abnormal patterns of vasodilation after neural activation. Vasculature changes such as increased tortuosity and rigidity of the blood vessels can also affect hemodynamic responses. Neurological disease also triggers inflammation and gliosis in the injured regions of the brain with activation and proliferation of glial cells (including astrocytes, microglia) (28-30).

Following an ischemic stroke, BBB disruption occurs acutely and triggers an inflammatory cascade of events, which can exacerbate brain damage (31). Particularly in diabetic stroke, both in humans and animal studies, it has been shown that there is exacerbated BBB leakage, white matter damage, vascular damage, and inflammatory responses that contribute to increased mortality and poor long term functional recovery (32-36). Exercise raises metabolic demand and the brain receives higher CBF from an elevated cardiac output (37,38). However, in type 2 diabetes, due to decreased vasodilation capacity, patients suffer from a diminished ability to increase cardiac output when responding to the increased oxygenation demands during exercise, often resulting in the perception of exertion (39).

Neurovascular uncoupling can also result from other conditions such as aging, hypertension, diabetes, traumatic brain injury, and dementia. The severity of hypertension proportionately decreases CBF and induces oxygen metabolism impairment (40). In the elderly, hypertension-induced CBF decrease can induce white matter lesions and lead to cognitive impairment and vascular dementia (40,41). Aging impairs dynamic CBF regulation and leads to cognitive dysfunction. It induces deficiency in circulating insulin-like growth factor-1, which hinders neurovascular coupling by 1) NO, due to endothelial dysfunction and 2) glutamate, due to astrocytic dysfunction, the end result being cognitive decline (42). Hence, neurovascular uncoupling plays a key role in the pathophysiology of several diseases, and developing therapeutic strategies that maintain or restore neurovascular coupling is imperative.

## Glymphatic dysfunction in neurological disease state

While on the one hand, neurovascular coupling is essential to supply the brain with oxygen and nutrients for its metabolic activity, on the other hand, a waste clearance system is vital to remove metabolic wastes and prevent toxic buildup. Recently, the glymphatic pathway has emerged as a functional and effective waste clearance pathway for the brain (43,44). This pathway consists of CSF influx into the brain parenchyma via para-arterial spaces, exchange of solutes (soluble proteins, waste products, metabolic wastes, and excess extracellular fluid) with the interstitial fluid (ISF), and clearance along para-venous spaces (43,44).While the cerebrospinal fluid (CSF) influx is driven by arterial pulsation, the exchange of solutes with the ISF and fluid movement through the parenchyma is driven by convective bulk flow rather than diffusion (43,44). The exchange of solutes between the CSF and the ISF occurs during sleep, when the cortical interstitial space increases by more than 60% and provides a low resistance path for the movement of CSF and ISF in the brain parenchyma (45).Water homeostasis is mediated by integral membrane pore proteins called aquaporins, which transport and regulate water movement in the brain. Aquaporin-4 (AQP-4) is predominantly present in astrocytic endfeet near capillaries and in cells lining the ventricles, which are key sites for water movement between the cellular, vascular, and ventricular compartments (46).The continuous AQP-4 expressing astrocytic endfeet lining the cerebral blood vessels create a low resistance para-vascular channel for the movement of CSF (47). Post-injury reduction of AQP-4 expression has been associated with exacerbated glymphatic system dysfunction (44), and AQP-4 knockout mice exhibited slowed CSF influx and ~ 70% reduction in ISF solute clearance, indicating that the AQP-4 water channel mediates/facilitates the glymphatic pathway (47). To control water influx into the brain, a sudden decrease in AQP-4 has been observed in regions of vascular damage post ischemia (48). Loss of AQP-4 polarization from astrocytic endfeet has been reported in cerebrovascular disorders and commonly occurs alongside an increase in AQP-4 in the parenchyma (49,50). Proper functioning of the water channel and glymphatic system is also central to the formation and resolution of edema after brain injury (43,44). Glymphatic dysfunction has been reported in neurological disease states such as stroke, traumatic brain injury, and Alzheimer disease (6,44,51). In Alzheimer disease, glymphatic impairment has emerged as a piece of the disease pathology puzzle. The amyloid beta (Aβ) peptide, which typically accumulates for years preceding Alzheimer dementia, is also produced by the normal brain and is present in the circulating blood and CSF (52). However, unlike the healthy brain that is able to clear Aβ via glymphatic drainage, in Alzheimer disease there is a gradual Aβ buildup in the brain parenchyma and vascular structures leading to neurovascular uncoupling including CBF decrease, BBB disruption, and impairment of vasculature (53,54). Age-associated glymphatic dysfunction has been reported with decreased and delayed CSF penetration along paravascular pathways and pial surface (55). Since arterial pulsation is a key driving force for paravascular CSF-ISF exchange, impaired waste clearance in the aging brain may be attributed to decreased ISF flow as a result of aging-induced vascular abnormalities, such as increased vascular stiffness, decreased vascular tone, decreased vessel wall pulsatility, and age-associated cardiac abnormalities (53,55). The mechanics and importance of the glymphatic system in several cerebrovascular disorders are still being unraveled and investigations of therapeutic strategies that can protect or restore its integrity are warranted.
